# Potential role of miR-155-5p in fat deposition and skeletal muscle development of chicken

**DOI:** 10.1042/BSR20193796

**Published:** 2020-06-03

**Authors:** Sifan Xu, Yang Chang, Guanxian Wu, Wanting Zhang, Chaolai Man

**Affiliations:** College of Life Science and Technology, Harbin Normal University, Harbin 150001, P. R. China

**Keywords:** chicken, clone, expression, fat, miR-155-5p, skeletal muscle

## Abstract

miR-155 has multiple functions in many physiological and pathological processes. However, little is known about the expression characteristics of avian miR-155. In the present study, partial pri-miR-155 sequences were cloned from AA^+^ broiler, Sanhuang broiler and Hy-Line Brown layer, respectively. Stem–loop qRT-PCR was performed to detect the miR-155-5p spatiotemporal expression profiles of each chicken breed, and the target genes of miR-155-5p were predicted in Gene Oncology (GO) and Kyoto Encyclopedia of Genes and Genomes (KEGG) pathways. The results showed that the partial pri-miR-155 sequences of different breeds of chicken were high conserved. The expression patterns of miR-155-5p between broiler and layer were basically similar, and miR-155-5p was expressed highly in immune related tissues (spleen, thymus and bursa). In the same old chicken (14 days old), miR-155-5p expression activity of fat tissue all had higher level in the three chicken breeds, but the expression activities in skeletal muscle of broilers were significantly lower than that of layer (*P*<0.05). In different development stages of Hy-Line Brown layer, miR-155-5p expression activities in skeletal muscle of 14-day-old and 10-month-old layers were significantly lower than that of 24-month-old layer (*P*<0.05). Fat related target genes (*ACOX1, ACOT7, FADS1, SCD* and *HSD17B12*) and skeletal muscle related target genes (*CCNT2, DMD, CFL2, MAPK14, FLNB, ZBTB18* and *CDK5*) of miR-155-5p were predicted, respectively. The results indicate that miR-155-5p may be an important factor inhibiting the fat deposition and skeletal muscle development in chicken.

## Introduction

MicroRNAs (miRNAs) are a class of endogenous non-coding regulatory RNAs, which are 18–23 nucleotides in length and negatively regulate gene expression at the posttranscriptional levels. miRNAs play critical roles in many biological processes, including cell proliferation, differentiation, development and functions [1–4]. Numerous studies have shown that miR-155 plays important roles in the immune system and can affect the activation of B, T cell and lymphocytes through controlling manifold regulatory roles [5–9]. Interestingly, the emerging evidences of miR-155 role in the development of skeletal muscle and adipogenesis have been noticed recently. MiR-155 can repress the expression of MEF2A (a member of the myogenic enhancer factor 2 family of transcription factors) and osteoglycin (Ogn, an important component of the skeletal muscle secretome), and inhibit proliferation and differentiation of myoblast cells [10,11]. MiR-155 also regulates the expression of Olfactomedin-like 3 (OLFML3), which may affect prenatal skeletal muscle development in pig [12]. Moreover, miR-155 can inhibit adipogenesis by directly targeting CCAAT/enhancer-binding protein β (C/EBPβ), cAMP-response element binding protein (CREB) and peroxisome proliferator-activated receptor gamma (PPARγ) [13–15]. MiR-155 also can inhibit brown adipose tissue formation and reduce a brown adipocyte-like phenotype (‘browning’) in white adipocytes in mice. MiR-155 and C/EBPβ co-regulate the development of brown and beige fat cells via a bistable circuit [16].

Although miR-155 has multiple functions in many biological and pathological processes, the possible physiological roles of miR-155 in various tissues of chicken remain unknown. Now, the poultry industry is facing several problems, including an increase in disease incidence, abundant fat deposits and breed improvement. Therefore, an in-depth analysis of the miR-155 tissue expression characteristics in different chicken breeds could provide valuable references to address these issues. It is generally known that there are wide differences on meat quantity and flavor, growth rate and disease resistance between broiler and layer. In the present study, three chicken breeds (AA^+^ broiler, Sanhuang broiler and Hy-Line Brown layer) were selected as study objects. AA^+^ chicken and Hy-Line Brown chicken are classical broiler and layer, respectively. Sanhuang chicken, as one breed of broiler, has good meat quantity and flavor, and its production traits are between broiler and layer. We cloned and analyzed partial pri-miR-155 sequences from the three breeds of chickens, then analyzed the expression profiles of miR-155-5p and predicted its target genes. The results can provide positive references for further understanding the function and possible application of miR-155-5p in chicken.

## Materials and methods

### Animals and samples collection

Sanhuang broiler (14 days old), AA^+^ broiler (14 days old) and different days old Hy-Line Brown layers (14 days old, 10 months old and 26 months old) were used as animal models. Tissue samples were obtained from the heart, liver, spleen, lung, kidney, thymus, large intestine, small intestine, muscular stomach, glandular stomach, skeletal muscle, skin, brain, fat and bursa of all chicken, then frozen in liquid nitrogen and stored at −80°C.

### RNA isolation and cloning of partial pri-miR-155 sequence

Total RNA was isolated from different tissue samples using TRIzol reagent (Sigma-Aldrich, St. Louis, MO, U.S.A.) according to the manufacturer protocols. RNA samples were digested with DNase I (TaKaRa, Dalian) for 1 h at 37°C to remove genomic DNA. RNA concentrations were measured with ultraviolet spectrophotometer. One microgram of total RNA from each sample was reversely transcribed into cDNA using a FSK-100 RT reagent Kit (TOYOBO, Shanghai) according to the manufacturer instructions.

Partial pri-miR-155 sequences of the three breeds of chickens were cloned using PCR with the cDNA from liver tissue above. The 50 μl of reaction system contained 1.0 μl cDNA (20 ng/μl), 4.0 μl dNTPs (2.5 mM) (TaKaRa, Dalian), 5 μl 10×Pyrobest buffer II (TaKaRa, Dalian), 2.5 μl 10 pM forward primer (5′-GTGCCCTTAACTTAGACCACATT-3′), 2.5 μl 10 pM reverse primer (5′-TCTAGAGTTCTTCTGTAGGCTGT-3′), 0.5 μl high-fidelity DNA polymerase (TaKaRa, Dalian) and 34.5 μl sterile water. The primers for chicken pri-miR-155 sequence isolation were designed based on the sequence from *Gallus gallus* (GenBank accession no. DN830517). The PCR program started with a 94°C for 4 min, and 30 cycles of 94°C/45 s, 56°C/45 s, 72°C/30 s, then 72°C extension for 10 min, finally 4°C to terminate the reaction. PCR products of the expected size of 263 bp were obtained. The PCR products were cloned into pMD18-T vector (TaKaRa, Dalian) and constructed recombinant vector pMD18-T-155. At least three independent recombinant plasmid clones from each breed were sequenced by Sangon Biotech (Shanghai) Co., Ltd. The partial pri-miR-155 sequences of the three chicken breeds were identical and have been deposited in the GenBank database and assigned GenBank accession no. KU365165.

### Bioinformatic analysis of miR-155

Homologous comparison and phylogenetic analysis of pre-miR-155 sequences from 23 different species were performed with DNAMAN version 7 software (http://www.lynnon.com) and MEGA7 (https://www.megasoftware.net/), respectively.

To analyze the miR-155-5p target gene, we obtained the target genes that had been experimentally verified from miRWalk 2.0 (http://zmf.umm.uni-heidelberg.de/apps/zmf/mirwalk2/). Then, Gene Oncology (GO) and Kyoto Encyclopedia of Genes and Genomes (KEGG) enrichment analysis of the target genes (*P*<0.05) were finished by DAVID v6.8 (https://david.ncifcrf.gov/).

### Stem–loop quantitative real-time PCR (qRT-PCR) for tissue expression profile analysis

MiR-155-5p expression in the different tissues was analyzed by stem–loop qRT-PCR. The chicken *U6* gene (GenBank accession no. NR004394) was selected as the internal control. The cDNAs of miR-155-5p and *U6* were produced with the stem-loop primer 5′-CTCAACTGGTGTCGTGGAGTCGGCAATTCAGTTGAGCCCCTATC-3′ and an anchored-oligo (dT)17 primer using the same method as above, respectively. On aliquots of the cDNA, qRT-PCR was performed simultaneously with miR-155-5p primers (5′-ACACTCCAGCTGGGTTAATGCTAATCGTGA-3′ and 5′-TGGTGTCGTGGAGTCG-3′) and *U6* primers (5′-CTCGCTTCGGCAGCACA-3′ and 5′-AACGCTTCACGAATTTGCGT-3′), respectively. The dose and reaction procedure of the qRT-PCR system of the miR-155-5p and *U6* were the same. The 20 μl reaction system contained 2 μl cDNA of each tissue (100 ng/μl), 10 pM each oligonucleotide primer, 10 μl THUNDERBIRD SYBR® qPCR Mix (TOYOBO, Shanghai), 10 μl 50×ROX reference dye (TOYOBO, Shanghai) and finally added sterile water to volume 20 μl. The PCR program initially started with a 94°C denaturation for 5 min, followed by 40 cycles of 94°C/15 s and 56°C/45 s, finally 4°C to terminate the reaction.

### Statistical analysis

Three chickens were randomly selected for quantitative analysis in all groups and three technical repetitions per each chicken were carried out. The relative expression activities of miR-155-5p were calculated using 2^−ΔCt^ method. The data were analyzed by one-way analysis of variance (ANOVA) using IBM SPSS Statistics 20.0 software and GraphPad Prism v5.0 software (GraphPad Software, La Jolla, CA, U.S.A.).

## Results

### Sequence analysis

**Figure 1:**
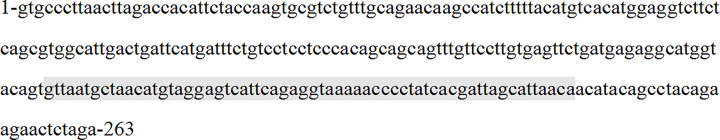
Partial pri-miR-155 sequence of chicken Nucleotide homology analysis of partial pri-miR-155 sequence of chicken using DNAMAN software (http://www.lynnon.com). The sequence of pre-miR-155 is shaded.

In the present study, partial pri-miR-155 sequences of the three chicken breeds (AA^+^ broiler, Sanhuang broiler and Hy-Line Brown layer) were cloned and sequenced. Homologous comparison of sequencing results revealed that the three partial pri-miR-155 sequences of each chicken breed shared the identical sequences with equal length (263 bp), and no deletion or insertion event was detected (Figure 1).

**Figure 2:**
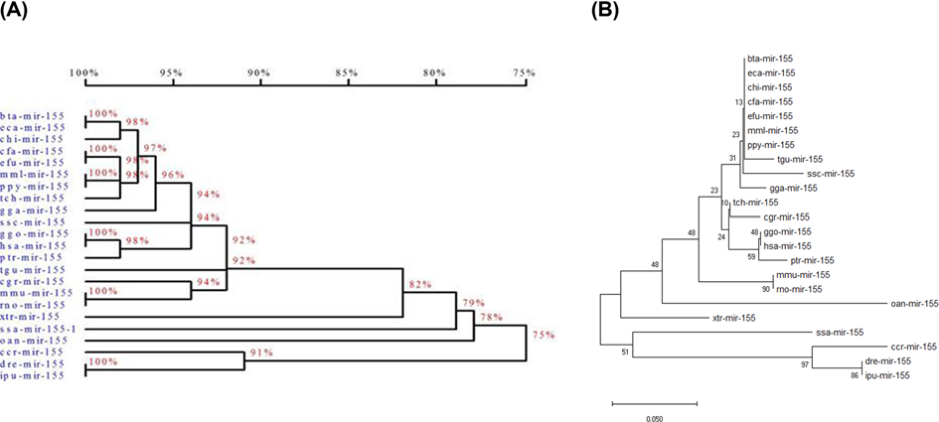
Homology and evolutionary analysis of multiple sequences of pre-miR-155 (**A**) Homology tree of multiple sequences of pre-miR-155. Homology tree of pre-miR-155 based on the nucleotide sequences. Analysis was done using the DNAMAN software (http://www.lynnon.com). The percentages on the branches represented homology. Interval range of scale corresponds to homology value of homologous tree. (**B**) Phylogenetic tree of multiple sequences of pre-miR-155. The tree was obtained by boot strap analysis with the neighbor-joining method; numbers on the branches represent bootstrap values for 1000 replications. aca-mir-155 (Anolis carolinensis, miRBase: MI0018764); bta-mir-155 (Bos taurus, miRBase: MI0009752); ccr-mir-155 (Cyprinus carpio, miRBase: MI0023336); cfa-mir-155 (Canis familiaris, miRBase: MI0008078); cgr-mir-155 (Cricetulus griseus, miRBase: MI0020415); chi-mir-155 (Capra hircus miRBase: MI0030643); dre-mir-155 (Danio rerio, miRBase: MI0002023); eca-mir-155 (Equus caballus, miRBase: MI0012927); efu-mir-155 (Eptesicus fuscus, miRBase: MI0028748); gga-mir-155 (Gallus gallus, miRBase: MI0001176); ggo-mir-155 (Gorilla gorilla, miRBase: MI0020768); hsa-mir-155 (Homo sapiens, miRBase: MI0000681); ipu-mir-155 (Ictalurus punctatus, miRBase: MI0024518); mml-mir-155 (Macaca mulatta, miRBase: MI0007645); mmu-mir-155 (Mus musculus, iRBase: MI0000177); oan-mir-155 (Ornithorhynchus anatinus, miRBase: MI0006775); ppy-mir-155 (Pongo pygmaeus, miRBase: MI0014843); ptr-mir-155 (Pan troglodytes, miRBase: MI0008554); rno-mir-155 (Rattus norvegicus, miRBase: MI0025509); ssa-mir-155-1 (Salmo salar, miRBase: MI0026520); ssc-mir-155 (Sus scrofa, miRBase: MI0015907); tch-mir-155 (Tupaia chinensis, miRBase: MI0031131); tgu-mir-155 (Taeniopygia guttata, miRBase: MI0013842); xtr-mir-155 (Xenopus tropicalis, miRBase: MI0004848).

Although the sequences of mature miR-155 from different species were highly conservative, the homology relationships of pre-miR-155 sequences among 23 different species showed differences. Homology analysis revealed that chicken pre-miR-155 was most similar to that of land mammals (over 92%) and birds, but not to that of amphibians and fish (below 82%) (Figure 2A). To evaluate the evolutionary relationships of chicken pre-miR-155 with other species, pre-miR-155 sequences from the 23 species were used for phylogenetic analysis using MEGA7 software. These pre-miR-155 sequences were basically divided into two subgroups (birds, mammals and amphibians were in one subgroup, and fish were in another subgroup), and chicken pre-miR-155 branched into the first group (Figure 2B).

### Stem–loop qRT-PCR analysis of miR-155-5p tissue distribution

**Figure 3:**
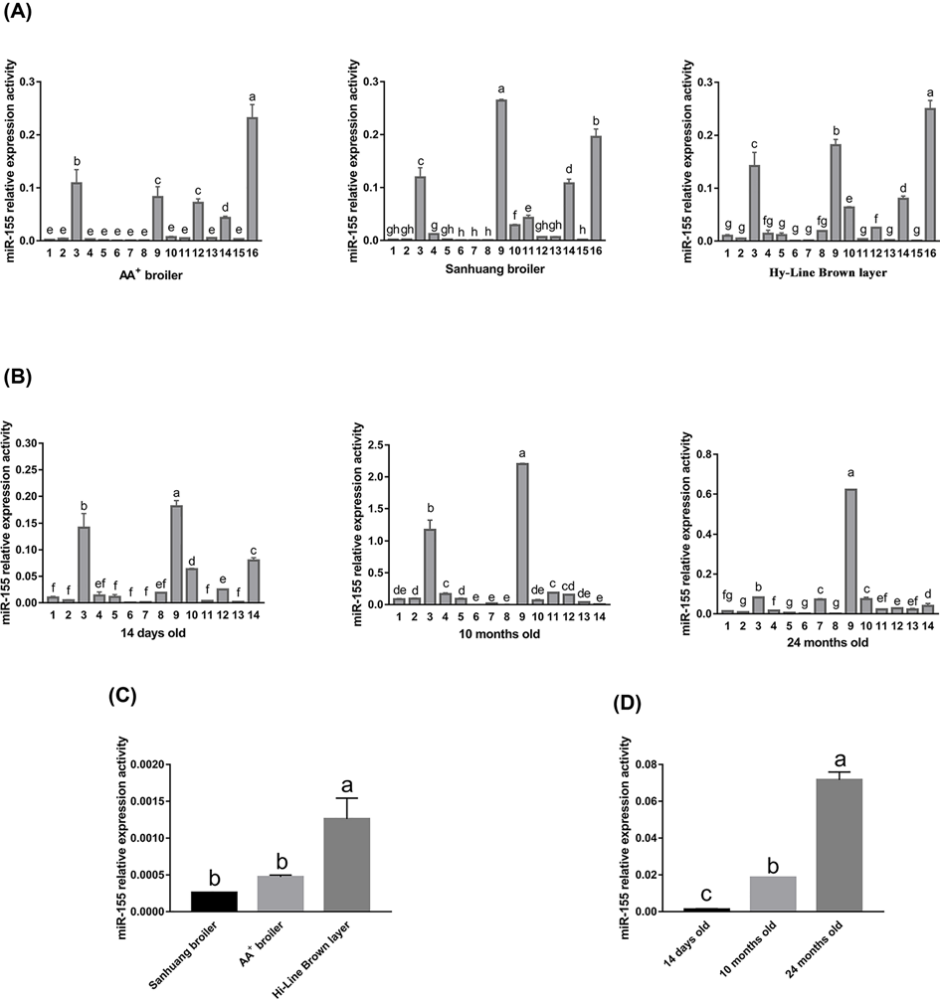
Expression patterns of miR-155-5p analyzed by stem-loop qRT-PCR (**A**) Expression distributions of miR-155-5p in three breeds of 14-day-old chicken. (**B**) Expression distributions of miR-155-5p in different developmental stages of Hy-Line Brown layer (14 days old, 10 months old and 24 months old). (**C**) Expression levels of miR-155-5p in skeletal muscle of three breeds of 14 days old chicken. (**D**) Expression levels of miR-155-5p in skeletal muscle of different developmental stages of Hy-Line Brown layer. 1 heart, 2 liver, 3 spleen, 4 lung, 5 kidney, 6 brain, 7 skeletal muscle, 8 muscular stomach, 9 thymus, 10 skin, 11 small intestine, 12 large intestine, 13 glandular stomach, 14 fat, 15 blood, 16 bursa. The internal reference gene is *U6*. The data are indicated as mean ± SD. Different letters (a−f) among different columns represent significant differences (*P*<0.05), while the same letters among different columns represent no significant differences.

The stem–loop qRT-PCR results showed that mature miR-155-5p was expressed in all tissues tested of the three chicken breeds, but the expression activity differed significantly between different tissues. According to the value of relative expression activity, we divide it into three levels. In AA^+^ broiler (14 days old), miR-155-5p was obvious differentially expressed in different tissues, with the high expression (the value of relative expression activity is over 0.2) in the bursa; medium expression (the value of relative expression activity is between 0.1 and 0.2) in the spleen; low expression (the value of relative expression activity is below 0.1) in the heart, liver, lung, kidney, brain, skeletal muscle, muscular stomach, thymus, skin, small intestine, large intestine, glandular stomach, fat and blood. In Sanhuang broiler (14 days old), the expression profile also presented significant differences, with the high expression in the thymus; medium expression in the spleen, bursa and fat; low expression in the other tissues. In Hy-Line Brown layer (14 days old), the expression profile also presented significant differences, with the high expression in the bursa; medium expression in the spleen and thymus; and low expression in the other tissues (Figure 3A).

In Hy-Line Brown layer (10 months old), the tissues of the high and medium expression were thymus and spleen, respectively, and other tissues were all the low expression. In Hy-Line Brown layer (24 months old), the tissue of high expression was only the thymus, and other tissues were all the low expression (Figure 3B). It is worth mentioning that the expression activities of miR-155-5p in fat tissues had higher level in the three 14-day-old chicken breeds (less fat deposition during this developmental stage), but decreased significantly in 10-month-old and 24-month-old layers (increasing fat deposition during this developmental stage) (Figure 3C). In addition, an interesting question that is easily overlooked is that the expression patterns of miR-155-5p in skeletal muscles of different chicken breeds (14 days old). Although all miR-155-5p had low expression activities in skeletal muscle, we found that the expression activities of miR-155-5p in 14-day-old broilers (AA^+^ broiler and Sanhuang broiler) were significantly lower than that of 14-day-old Hy-Line Brown layer. By comparing the expression activities of miR-155-5p in skeletal muscle at different developmental stages, it was found that miR-155-5p had low expression activity in 14-day-old and 10-month-old chickens, but increased in 24-month-old chicken significantly (*P*<0.05) (Figure 3D).

### Target gene prediction of miR-155-5p and analysis of GO and KEGG

**Figure 4:**
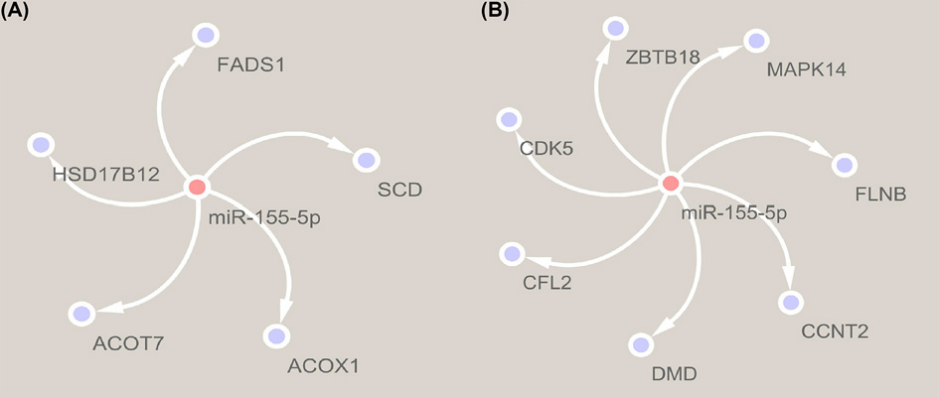
Target gene analysis of miR-155-5p (**A**) MiR-155-5p target genes associated with biosynthesis of unsaturated fatty acids (hsa01040). (**B**) MiR-155-5p target genes associated with skeletal muscle development (GO: 0007519). Acquisition of miR-155-5p target genes that had been experimentally verified using miRWalk 2.0 (http://zmf.umm.uni-heidelberg.de/apps/zmf/mirwalk2/). Then, Gene Oncology (GO) and Kyoto Encyclopedia of Genes and Genomes (KEGG) enrichment analysis of the target genes (P<0.05) by DAVID v6.8 (https://david.ncifcrf.gov/).

Five fat related target genes (*ACOX1, ACOT7, FADS1, SCD* and *HSD17B12*) of miR-155-5p were predicted, and which were identified in KEGG pathways (Figure 4A). Seven skeletal-muscle related genes (*CCNT2, DMD, CFL2, MAPK14, FLNB, ZBTB18* and *CDK5*) were identified in GO pathways (Figure 4B).

## Discussion

There are huge differences of growth characteristics between broiler and layer, such as growth speed, egg production and meat flavor, etc. In the present study, different chicken breeds were selected to analyze tissue expression profiles, which could provide conveniences for finding the functional differences of miR-155-5p in chicken.

The flanking ssRNA segments are critical for processing pri-miRNA and the cleavage site is determined mainly by the distance (approximately 11 bp) from the stem–ssRNA junction [17]. In the present study, the partial pri-miR-155 sequences were identical between the four breeds (including *Gallus gallus*, miRBase: MI0001176), so we speculated that the mechanisms of processing pri-miR-155 in different chicken breeds were identical. Homology analysis indicated that pre-miR-155 sequences were high conservative between different species. The phylogenetic analysis showed that chicken pre-miR-155 was in the middle site of evolution, and had closer genetic relationship with that of mammals and amphibians in comparison with fish as far as the evolutionary distance was concerned, which implied that chicken should be a better animal model to study the functions and mechanisms of miR-155.

According to the tissue expression profile analysis of the different breeds of the same age chicken (14 days old), we found that there was a fundamental similarity of miR-155-5p relative expression activity in different tissues among the three chicken breeds. For example, miR-155-5p expression activities were all the strongest expression in the immune-associated tissues (such as bursa, spleen and thymus). As we all know that the spleen, thymus and bursa are the major lymphoid organs of chicken immune system. The suitable explanation for this was that miR-155-5p expression might be related to the biological activities or development of these tissues. Moreover, the differences of miR-155-5p expression activity in immune-associated tissues between AA^+^ broiler, Sanhuang broiler and Hy-Line Brown layer might be associated with the different breeds.

In order to study whether the miR-155-5p expression is connected with development of chicken, the tissue expression profiles were analyzed in different development stages (14 days old, 10 months old and 24 months old) of the same breed (Hy-Line Brown layer). The expression activities of miR-155-5p in spleen and thymus were worthy of attentions. The expression activities of miR-155-5p in thymus always kept the strongest expression during different development stages, but the expression activities of miR-155-5p in spleen decreased significantly in 24 months old chicken. The possible reason is that the expression of miR-155-5p might be associated with organ function and characteristics at different developmental stages.

Studies have shown that miR-155-5p can inhibit adipogenesis [13–15]. Expression profile analysis showed that miR-155-5p expression activities in fat tissues had down-regulation trend during different developmental stages and several fat related genes were identified as the target genes of miR-155-5p in KEGG, which suggested that miR-155-5p might be an important negative regulator affecting fat deposition in chicken. Interestingly, miR-155-5p can also inhibit myoblast differentiation and skeletal muscle formation [10,11]. Expression activity analysis showed that miR-155-5p had higher expression activity in skeletal muscle of 14 days old layer than that of the same age broiler, and the expression activities were up-regulated with development in layer. In addition, we also identified several skeletal-muscle related target genes of miR-155-5p in GO pathway. So, we speculated that the low expression activity of miR-155-5p could promote skeletal muscle development in 14-day-old (especially in broilers) and 10-month-old chickens, while the skeletal muscle development of 24-month-old chick had basically stopped, so miR-155-5p expression activity was up-regulated significantly to inhibit skeletal muscle growth. However, the mechanisms of the miR-155-5p affecting fat deposition and skeletal muscle development in chicken remain to be further studied.

In conclusion, we cloned the partial pri-miR-155 sequences from broiler and layer, and found the partial pri-miR-155 sequences were high conserved in different chicken breeds. Expression profile analysis of miR-155-5p showed that layer and broiler had the basically similar expression pattern, and miR-155-5p was expressed highly in immune related tissues. The different expression activities of miR-155-5p suggested that miR-155-5p might be a key factor affecting the development of skeletal muscle and fat deposition of different breeds of chicken.

## Abbreviations

BDNF: brain-derived neurotrophic factor
C/EBPβ: CCAAT/enhancer-binding protein β
CREB: cAMP-response element binding protein
MEF2A: myogenic enhancer factor 2A
Ogn: osteoglycin
OLFML3: olfactomedin-like 3
PPARγ: peroxisome proliferator-activated receptor gamma
